# 4-(2,3-Di­hydro­thieno[3,4-*b*][1,4]dioxin-5-yl)aniline

**DOI:** 10.1107/S1600536814014093

**Published:** 2014-06-21

**Authors:** Lauren A. Mitchell, Bradley J. Holliday

**Affiliations:** a Department of Chemistry, The University of Texas at Austin, 105 E 24th Street, Stop A5300, Austin, Texas 78712, USA

**Keywords:** crystal structure

## Abstract

In the title mol­ecule, C_12_H_11_NO_2_S, the dioxane-type ring adopts a half-chair conformation. The thio­phene ring forms a dihedral angle of 12.53 (6)° with the benzene ring. In the crystal, N—H⋯O, hydrogen bonds link mol­ecules, forming chains along the *c-*axis direction. A weak intra­molecular C—H⋯O hydrogen bond is observed.

## Related literature   

For related structures, see: Chen *et al.* (2011 ▶); Riehn *et al.* (2000 ▶); Sotzing & Reynolds (1996 ▶). For the properties of 4-(2,3-di­hydro­thieno[3,4-*b*][1,4]dioxin-5-yl)aniline see: Trippé-Allard & Lacroix (2013 ▶).
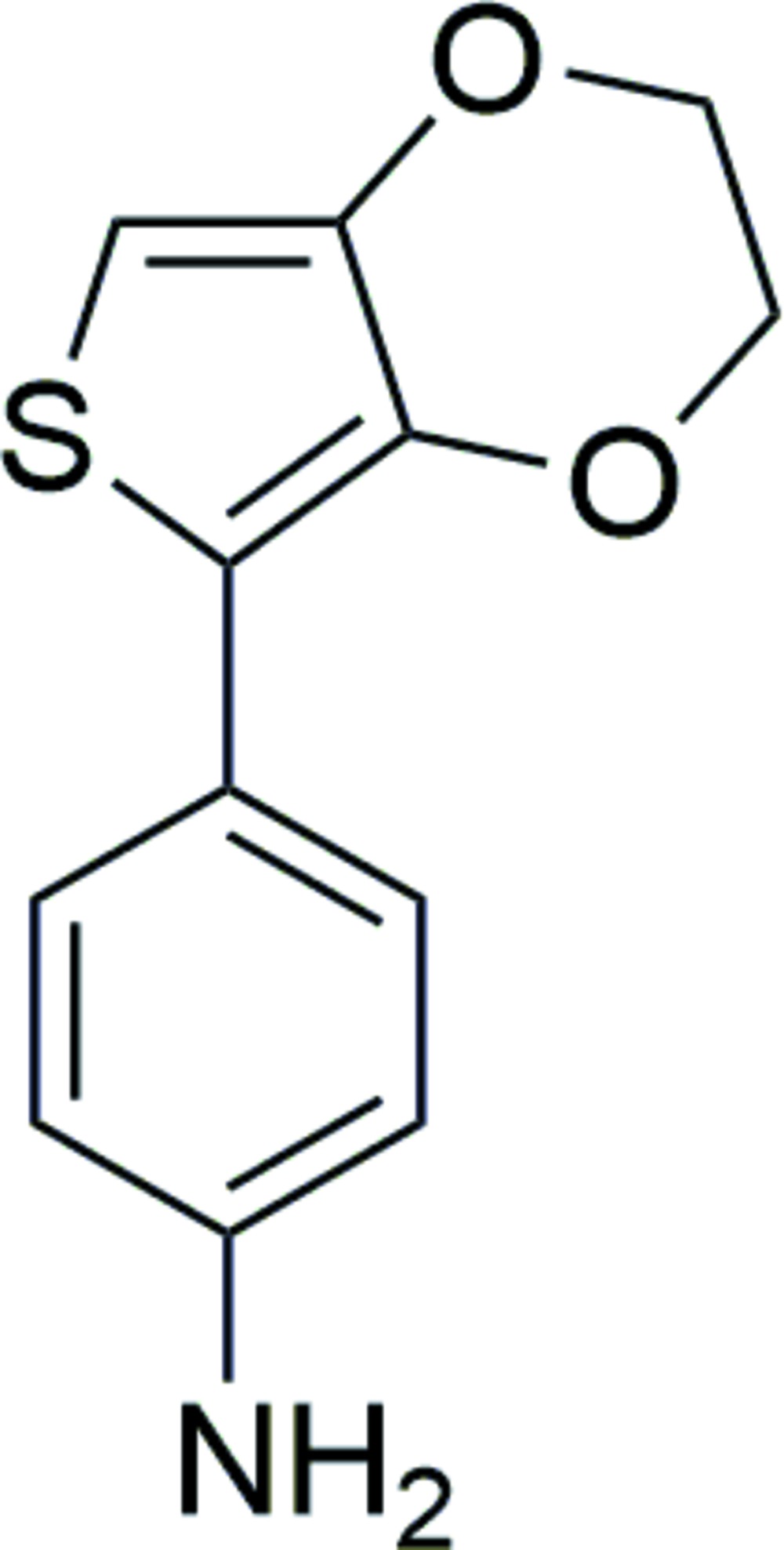


## Experimental   

### 

#### Crystal data  

C_12_H_11_NO_2_S
*M* *_r_* = 233.28
Orthorhombic, 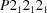

*a* = 6.9117 (6) Å
*b* = 7.0898 (6) Å
*c* = 21.4784 (16) Å
*V* = 1052.50 (15) Å^3^
*Z* = 4
Mo *K*α radiation
μ = 0.29 mm^−1^
*T* = 100 K
0.29 × 0.27 × 0.08 mm

#### Data collection  

Rigaku Saturn724+ diffractometer
Absorption correction: multi-scan (*ABSCOR*; Higashi, 2001 ▶) *T*_min_ = 0.858, *T*_max_ = 1.000
11854 measured reflections
1853 independent reflections
1812 reflections with *I* > 2σ(*I*)
*R*_int_ = 0.047

#### Refinement  

*R*[*F* ^2^ > 2σ(*F* ^2^)] = 0.029
*wR*(*F* ^2^) = 0.075
*S* = 0.86
1853 reflections
153 parameters
H atoms treated by a mixture of independent and constrained refinement
Δρ_max_ = 0.43 e Å^−3^
Δρ_min_ = −0.21 e Å^−3^
Absolute structure: Flack (1983 ▶), 743 Friedel pairs
Absolute structure parameter: 0.03 (8)

### 

Data collection: *CrystalClear* (Rigaku, 2008 ▶); cell refinement: *CrystalClear*; data reduction: *CrystalClear*; program(s) used to solve structure: *SIR97* (Altomare *et al.*, 1999 ▶); program(s) used to refine structure: *SHELXL97* (Sheldrick, 2008 ▶) within *WinGX* (Farrugia, 2012 ▶); molecular graphics: *ORTEP-3 for Windows* (Farrugia, 2012 ▶), *POV-RAY* (Cason, 2004 ▶) and *Mercury* (Macrae *et al.*, 2008 ▶); software used to prepare material for publication: *SHELXL97* and *publCIF* (Westrip, 2010 ▶).

## Supplementary Material

Crystal structure: contains datablock(s) I. DOI: 10.1107/S1600536814014093/lh5708sup1.cif

Structure factors: contains datablock(s) I. DOI: 10.1107/S1600536814014093/lh5708Isup2.hkl

Supporting information file. DOI: 10.1107/S1600536814014093/lh5708Isup3.cml

CCDC reference: 1008614

Additional supporting information:  crystallographic information; 3D view; checkCIF report

## Figures and Tables

**Table 1:** Hydrogen-bond geometry (Å, °)

*D*—H⋯*A*	*D*—H	H⋯*A*	*D*⋯*A*	*D*—H⋯*A*
N1—H10*A*⋯O1^i^	0.88 (3)	2.52 (3)	3.352 (2)	160 (2)
C8—H8⋯O2	0.93	2.36	2.998 (2)	126

Symmetry code: (i) 
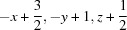
.

**Fig. 1.**
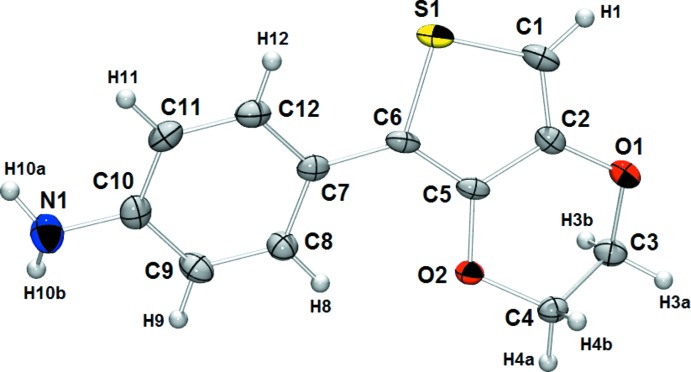
Molecular structure of title compound. Ellipsoids are drawn at the 50% probability level.

**Fig. 2.**
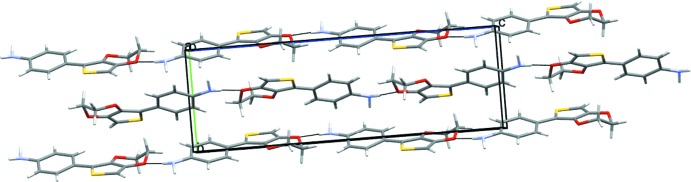
Part of the crystal structure viewed along the b axis. Thin black lines indicate N—H···O hydrogen bonds.

## supplementary crystallographic information

### S1. Comment

The title compound is composed of an aniline moiety with a 3,4-ethylenedioxythiophene group appended at the 4-position, see Fig. 1. It has been used in the development of π-conjugated oligomers, which have low HOMO-LUMO gaps and are easily oxidized at low potentials, making them potential materials for photovoltaics and other optoelectronic applications (Trippé-Allard & Lacroix, 2013). The geometry of the ethylenedioxythiophene moiety is similar to other ethylenedioxythiophene containing compounds reported in the literature, which includes the six-membered dioxane-type ring in the half-chair conformation (Chen *et al.*, 2011; Sotzing & Reynolds, 1996; Riehn *et al.*, 2000). The dihedral angle between the thiophene and benzene rings is 12.53 (6)°. In the crystal, N1—H10A···O1^i^ hydrogen bonds link molecules into chains along the *c* axis (Fig. 2). A weak intramolecular C—H···O hydrogen bond is also observed.

### S2. Experimental

To a solution of dry toluene under N_2_ was added tributyl(2,3-dihydrothieno[3,4-*b*][1,4]dioxin-5-yl)stannane (21.4 g, 49.5 mmol), 4-iodonitrobenzene (7.7 g, 30.9 mmol), *trans*-dichloro*bis*(triphenylphosphine) palladium (II) (0.3 g, 0.5 mmol), and copper (I) chloride (0.2 g, 1.1 mmol). The solution was refluxed at 383 K overnight. The black solution was exposed to atmosphere and conc. under reduced pressure. The solid was dissolved in dichloromethane and filtered over a bed of silica. The filtrate was conc. and recycrystallized in a dichloromethane/hexanes mixture to yield a bright yellow solid. The isolated yellow solid was added to a round bottom and dissolved in tetrahydrofuran (THF). Charcoal (8.39 g) and 5 ml of H_2_O was added and the mixture was heated to 323 K. Sodium borohydride (2.66 g, 70.5 mmol) was added in four portions over 1 hr. The reaction was heated for an additional 30 min after the last addition. The mixture was cooled to room temp. and filtered, washing with THF. The solution was concentrated then re-dissolved in CH_2_Cl_2_ and washed with H_2_O. The organic layer was concentrated to a third the original volume and mixed with an equal volume of hexanes. The solution was left standing overnight at 273 K and the orange crystals that precipitated were collected by vacuum filtration (4.63 g, 64% yield). These crystals were found suitable for X-ray diffraction. m.p. 376 K. ^1^H NMR (300 MHz, CDCl_3_): δ 7.51 (dt, *J* = 8.7, *J* = 2.1, 2H), 6.66 (dt, *J* = 8.7, *J* = 2.4, 2H), 6.19 (s, 1H), 4.25 – 4.18 (m, 4H), 3.64 (b, 2H); ^13^C{^1^H} NMR (75 MHz, CDCl_3_): δ 145.2, 142.1, 136.6, 127.2, 123.5, 117.9, 115.0, 95.5, 64.5, 64.4; Anal Calcd for C_12_H_11_NO_2_S: C, 61.78; H, 4.75; N, 6.00. Found: C, 61.67; H, 4.07; N, 5.90.

### S3. Refinement

The amine H atoms were located in a difference Fourier map and both positional and isotropic displacement parameters were refined. All other H atoms were positioned geometrically and refined using a riding model, with C—H = 0.93–0.97 Å and with *U*_iso_(H) = 1.2 times *U*_eq_(C).

### Crystal data

**Table:**

C_12_H_11_NO_2_S	*F*(000) = 488
*M**_r_* = 233.28	*D*_x_ = 1.472 Mg m^−^^3^
Orthorhombic, *P*2_1_2_1_2_1_	Mo *K*α radiation, λ = 0.71075 Å
Hall symbol: P 2ac 2ab	Cell parameters from 2964 reflections
*a* = 6.9117 (6) Å	θ = 2.9–28.2°
*b* = 7.0898 (6) Å	µ = 0.29 mm^−^^1^
*c* = 21.4784 (16) Å	*T* = 100 K
*V* = 1052.50 (15) Å^3^	Plate, orange
*Z* = 4	0.29 × 0.27 × 0.08 mm

### Data collection

**Table:**

Rigaku Saturn724+ diffractometer	1853 independent reflections
Radiation source: fine-focus sealed tube	1812 reflections with *I* > 2σ(*I*)
Graphite monochromator	*R*_int_ = 0.047
Detector resolution: 28.5714 pixels mm^-1^	θ_max_ = 25.0°, θ_min_ = 3.0°
profile data from ω scans	*h* = −8→8
Absorption correction: multi-scan (*ABSCOR*; Higashi, 2001)	*k* = −8→8
*T*_min_ = 0.858, *T*_max_ = 1.000	*l* = −22→25
11854 measured reflections	

### Refinement

**Table:**

Refinement on *F*^2^	Secondary atom site location: difference Fourier map
Least-squares matrix: full	Hydrogen site location: inferred from neighbouring sites
*R*[*F*^2^ > 2σ(*F*^2^)] = 0.029	H atoms treated by a mixture of independent and constrained refinement
*wR*(*F*^2^) = 0.075	*w* = 1/[σ^2^(*F*_o_^2^) + (0.0543*P*)^2^ + 0.6318*P*] where *P* = (*F*_o_^2^ + 2*F*_c_^2^)/3
*S* = 0.86	(Δ/σ)_max_ = 0.001
1853 reflections	Δρ_max_ = 0.43 e Å^−^^3^
153 parameters	Δρ_min_ = −0.21 e Å^−^^3^
0 restraints	Absolute structure: Flack (1983), 743 Friedel pairs
Primary atom site location: structure-invariant direct methods	Absolute structure parameter: 0.03 (8)

### Special details

**Table:**

Geometry. All e.s.d.'s (except the e.s.d. in the dihedral angle between two l.s. planes) are estimated using the full covariance matrix. The cell e.s.d.'s are taken into account individually in the estimation of e.s.d.'s in distances, angles and torsion angles; correlations between e.s.d.'s in cell parameters are only used when they are defined by crystal symmetry. An approximate (isotropic) treatment of cell e.s.d.'s is used for estimating e.s.d.'s involving l.s. planes.
Refinement. Refinement of *F*^2^ against ALL reflections. The weighted *R*-factor *wR* and goodness of fit *S* are based on *F*^2^, conventional *R*-factors *R* are based on *F*, with *F* set to zero for negative *F*^2^. The threshold expression of *F*^2^ > σ(*F*^2^) is used only for calculating *R*-factors(gt) *etc*. and is not relevant to the choice of reflections for refinement. *R*-factors based on *F*^2^ are statistically about twice as large as those based on *F*, and *R*- factors based on ALL data will be even larger.

### Fractional atomic coordinates and isotropic or equivalent isotropic displacement parameters (Å^2^)

**Table:**

	*x*	*y*	*z*	*U*_iso_*/*U*_eq_	
S1	0.53914 (6)	0.63412 (6)	0.83637 (2)	0.02511 (15)	
O1	0.73741 (18)	0.56761 (18)	0.66914 (6)	0.0229 (3)	
O2	1.02695 (18)	0.47874 (17)	0.76297 (6)	0.0203 (3)	
N1	1.1401 (3)	0.4133 (3)	1.07137 (9)	0.0332 (4)	
C1	0.5329 (3)	0.6408 (3)	0.75642 (9)	0.0257 (4)	
H1	0.4267	0.6801	0.7332	0.031*	
C2	0.7019 (3)	0.5818 (2)	0.73164 (9)	0.0199 (4)	
C3	0.9069 (3)	0.4540 (3)	0.65674 (9)	0.0238 (4)	
H3A	0.9447	0.4687	0.6135	0.029*	
H3B	0.8767	0.3220	0.6638	0.029*	
C4	1.0715 (3)	0.5118 (3)	0.69824 (8)	0.0207 (4)	
H4A	1.1865	0.4411	0.6870	0.025*	
H4B	1.0988	0.6447	0.6921	0.025*	
C5	0.8432 (2)	0.5355 (2)	0.77795 (8)	0.0182 (4)	
C6	0.7780 (2)	0.5557 (2)	0.83812 (9)	0.0196 (4)	
C7	0.8775 (3)	0.5263 (2)	0.89751 (9)	0.0199 (4)	
C8	1.0548 (3)	0.4311 (2)	0.90137 (9)	0.0225 (4)	
H8	1.1154	0.3915	0.8650	0.027*	
C9	1.1417 (3)	0.3948 (3)	0.95825 (9)	0.0262 (4)	
H9	1.2593	0.3312	0.9595	0.031*	
C10	1.0543 (3)	0.4530 (3)	1.01393 (9)	0.0250 (4)	
C11	0.8850 (3)	0.5570 (3)	1.01015 (9)	0.0266 (4)	
H11	0.8296	0.6041	1.0464	0.032*	
C12	0.7970 (3)	0.5920 (3)	0.95357 (9)	0.0257 (4)	
H12	0.6823	0.6605	0.9526	0.031*	
H10A	1.065 (4)	0.415 (3)	1.1041 (11)	0.035 (6)*	
H10B	1.215 (4)	0.315 (4)	1.0685 (12)	0.042 (7)*	

### Atomic displacement parameters (Å^2^)

**Table:**

	*U*^11^	*U*^22^	*U*^33^	*U*^12^	*U*^13^	*U*^23^
S1	0.0134 (2)	0.0283 (3)	0.0336 (3)	0.0022 (2)	0.0016 (2)	−0.0034 (2)
O1	0.0188 (6)	0.0237 (6)	0.0262 (7)	0.0012 (5)	−0.0045 (6)	−0.0017 (5)
O2	0.0152 (6)	0.0226 (6)	0.0230 (6)	0.0032 (6)	0.0014 (5)	0.0021 (5)
N1	0.0385 (11)	0.0316 (10)	0.0295 (11)	0.0059 (9)	−0.0036 (9)	0.0010 (8)
C1	0.0154 (8)	0.0235 (8)	0.0382 (11)	0.0007 (9)	−0.0046 (8)	−0.0025 (8)
C2	0.0185 (9)	0.0146 (8)	0.0264 (10)	−0.0029 (7)	−0.0037 (7)	0.0002 (7)
C3	0.0210 (9)	0.0215 (9)	0.0289 (10)	0.0005 (8)	0.0002 (7)	−0.0030 (8)
C4	0.0191 (9)	0.0185 (8)	0.0245 (10)	0.0006 (7)	0.0026 (8)	0.0001 (7)
C5	0.0123 (8)	0.0121 (8)	0.0302 (10)	−0.0009 (7)	−0.0002 (7)	−0.0007 (7)
C6	0.0121 (8)	0.0146 (8)	0.0322 (10)	−0.0004 (7)	0.0012 (8)	0.0009 (8)
C7	0.0181 (9)	0.0144 (8)	0.0271 (10)	−0.0032 (7)	0.0029 (7)	0.0014 (7)
C8	0.0225 (9)	0.0204 (8)	0.0244 (9)	0.0030 (8)	0.0000 (8)	−0.0019 (7)
C9	0.0233 (9)	0.0226 (9)	0.0327 (11)	0.0053 (8)	−0.0040 (8)	−0.0028 (8)
C10	0.0284 (10)	0.0202 (8)	0.0264 (10)	−0.0053 (9)	−0.0015 (8)	0.0030 (8)
C11	0.0263 (10)	0.0288 (10)	0.0247 (10)	−0.0014 (9)	0.0074 (8)	−0.0011 (8)
C12	0.0202 (9)	0.0243 (9)	0.0327 (11)	0.0022 (8)	0.0048 (8)	0.0027 (8)

### Geometric parameters (Å, º)

**Table:**

S1—C1	1.718 (2)	C4—H4A	0.9700
S1—C6	1.7424 (18)	C4—H4B	0.9700
O1—C2	1.368 (2)	C5—C6	1.376 (3)
O1—C3	1.446 (2)	C6—C7	1.464 (3)
O2—C5	1.370 (2)	C7—C8	1.402 (3)
O2—C4	1.443 (2)	C7—C12	1.406 (3)
N1—C10	1.397 (3)	C8—C9	1.385 (3)
N1—H10A	0.87 (3)	C8—H8	0.9300
N1—H10B	0.87 (3)	C9—C10	1.402 (3)
C1—C2	1.350 (3)	C9—H9	0.9300
C1—H1	0.9300	C10—C11	1.385 (3)
C2—C5	1.432 (3)	C11—C12	1.382 (3)
C3—C4	1.503 (3)	C11—H11	0.9300
C3—H3A	0.9700	C12—H12	0.9300
C3—H3B	0.9700		
			
C1—S1—C6	93.10 (9)	O2—C5—C6	123.69 (16)
C2—O1—C3	111.52 (14)	O2—C5—C2	122.42 (16)
C5—O2—C4	112.11 (13)	C6—C5—C2	113.89 (16)
C10—N1—H10A	117.2 (17)	C5—C6—C7	130.51 (16)
C10—N1—H10B	110.5 (17)	C5—C6—S1	108.87 (14)
H10A—N1—H10B	115 (2)	C7—C6—S1	120.60 (14)
C2—C1—S1	111.33 (15)	C8—C7—C12	117.05 (17)
C2—C1—H1	124.3	C8—C7—C6	122.06 (16)
S1—C1—H1	124.3	C12—C7—C6	120.89 (17)
C1—C2—O1	124.39 (17)	C9—C8—C7	121.37 (18)
C1—C2—C5	112.78 (17)	C9—C8—H8	119.3
O1—C2—C5	122.83 (16)	C7—C8—H8	119.3
O1—C3—C4	110.61 (14)	C8—C9—C10	120.74 (18)
O1—C3—H3A	109.5	C8—C9—H9	119.6
C4—C3—H3A	109.5	C10—C9—H9	119.6
O1—C3—H3B	109.5	C11—C10—N1	121.13 (19)
C4—C3—H3B	109.5	C11—C10—C9	118.06 (18)
H3A—C3—H3B	108.1	N1—C10—C9	120.76 (19)
O2—C4—C3	111.42 (15)	C12—C11—C10	121.27 (18)
O2—C4—H4A	109.3	C12—C11—H11	119.4
C3—C4—H4A	109.3	C10—C11—H11	119.4
O2—C4—H4B	109.3	C11—C12—C7	121.31 (18)
C3—C4—H4B	109.3	C11—C12—H12	119.3
H4A—C4—H4B	108.0	C7—C12—H12	119.3
			
C6—S1—C1—C2	1.54 (14)	C2—C5—C6—S1	−0.29 (19)
S1—C1—C2—O1	178.30 (13)	C1—S1—C6—C5	−0.68 (13)
S1—C1—C2—C5	−2.0 (2)	C1—S1—C6—C7	177.95 (14)
C3—O1—C2—C1	−164.18 (17)	C5—C6—C7—C8	−13.8 (3)
C3—O1—C2—C5	16.1 (2)	S1—C6—C7—C8	167.89 (14)
C2—O1—C3—C4	−46.87 (19)	C5—C6—C7—C12	166.69 (19)
C5—O2—C4—C3	−44.06 (19)	S1—C6—C7—C12	−11.6 (2)
O1—C3—C4—O2	63.54 (19)	C12—C7—C8—C9	3.3 (3)
C4—O2—C5—C6	−166.28 (16)	C6—C7—C8—C9	−176.21 (18)
C4—O2—C5—C2	12.9 (2)	C7—C8—C9—C10	−0.1 (3)
C1—C2—C5—O2	−177.73 (15)	C8—C9—C10—C11	−3.7 (3)
O1—C2—C5—O2	2.0 (3)	C8—C9—C10—N1	178.79 (19)
C1—C2—C5—C6	1.5 (2)	N1—C10—C11—C12	−178.21 (19)
O1—C2—C5—C6	−178.78 (15)	C9—C10—C11—C12	4.3 (3)
O2—C5—C6—C7	0.5 (3)	C10—C11—C12—C7	−1.1 (3)
C2—C5—C6—C7	−178.74 (17)	C8—C7—C12—C11	−2.7 (3)
O2—C5—C6—S1	178.91 (13)	C6—C7—C12—C11	176.79 (17)

### Hydrogen-bond geometry (Å, º)

**Table:**

*D*—H···*A*	*D*—H	H···*A*	*D*···*A*	*D*—H···*A*
N1—H10*A*···O1^i^	0.88 (3)	2.52 (3)	3.352 (2)	160 (2)
C8—H8···O2	0.93	2.36	2.998 (2)	126

Symmetry code: (i) −*x*+3/2, −*y*+1, *z*+1/2.
